# *‘ … they just take one pill, so it is easy to use, more convenient … ’*: South African health care provider perspectives on a Dual Prevention Pill

**DOI:** 10.1080/17290376.2026.2678165

**Published:** 2026-06-03

**Authors:** Sanyukta Mathur, Siyanda Tenza, Lorna Begg, Lydia Mampuru, Irene V. Bruce, Mpho Moji, Nkosiphile Ndlovu, Krishnaveni Reddy, Thesla Palanee-Phillips, Barbara A. Friedland

**Affiliations:** a Social and Behavioral Research, Population Council, New York, NY, USA; b Wits RHI, University of the Witwatersrand, Johannesburg, South Africa; c Center for Biomedical Research, Population Council, New York, NY, USA; d Faculty of Health Sciences, School of Public Health, Wits RHI, University of the Witwatersrand, Johannesburg, South Africa; e Department of Epidemiology, School of Public Health, University of Washington, Seattle, WA, USA

**Keywords:** HIV prevention, contraception, healthcare providers, multipurpose prevention technologies, dual prevention pill, qualitative research

## Abstract

The Dual Prevention Pill (DPP) is a daily oral pill containing oral pre-exposure prophylaxis (PrEP) for protection against HIV and a combined oral contraceptive (COC) for the prevention of unintended pregnancy, currently under development. However, little is known about healthcare providers' (HCPs) perspectives on this potential new multipurpose prevention technology (MPT). A more granular understanding of HCP views about the DPP may support efforts to ensure equitable introduction of and access to this innovative technology. We conducted in-depth interviews with 17 HCPs working at family planning (FP) and HIV service sites in Johannesburg, South Africa. We used a qualitative framework analysis to examine HCP perspectives on potential end-users, the DPP concept and health system-related considerations. HCPs articulated that the DPP would meet the needs of their clients, contribute to more efficient and effective integrated sexual and reproductive health service delivery and meet broader community goals for the reduction in HIV infections and unintended pregnancies. HCPs noted concerns and potential challenges with the product’s daily dosing regimen, potential for exacerbated side effects (compared to oral PrEP or COCs taken alone) and potential ethical considerations about offering the DPP to adolescents. HCPs felt the DPP should be available through public health care facilities to maximise outreach and ensure appropriate care, and discussed community-based delivery options for expanded outreach. They also noted the need to address provider biases, particularly when serving adolescents, as well as the accompanying demand creation efforts with end-users and educational campaigns in the community. Understanding providers’ support for the DPP, as well as the potential challenges they foresee, should inform market introduction strategies, clinic counselling guidelines and provider training for the DPP. We underscore the need to engage with HCPs early (prior to product introduction) to understand local barriers and facilitators to ensure quality sustained rollout and support for MPTs.

## Introduction

Women and girls face overlapping risks of unintended pregnancy and HIV infection. Growing evidence indicates that women may prefer multipurpose prevention technologies (MPTs) for simultaneous prevention of HIV and unintended pregnancies to single-indication products (Beksinska et al., 2018; Friedland et al., 2023; Hynes, Sales, Sheth, Lathrop, & Haddad, 2018; IPSOS, 2014; Karim, Baxter, Frohlich, & Karim, 2014; van der Straten et al., 2018). Various MPTs are currently in development (IMPT, 2023), including a dual prevention pill (DPP) containing oral pre-exposure prophylaxis (PrEP) and a combined oral contraceptive (COC) (PrEPWatch, 2021). As PrEP and COCs are already approved and available, large efficacy trials will not be required, enabling an accelerated development pathway for the DPP. The DPP will likely be the first new MPT introduced since male and female condoms (Begg et al., 2021). There is increasing recognition of the importance of gathering end-user perspectives early in product development to inform product attributes and introduction strategies (Browne et al., 2023). However, less attention has been paid to healthcare provider perspectives (Henderson et al., 2023).

Healthcare providers (HCPs) are influential gatekeepers whose attitudes and clinical practices have an outsized influence on effective counselling and provision of new sexual and reproductive health products (Fataar, Zweigenthal, & Harries, 2022; Pleaner et al., 2023). Clinicians’ own biases and perspectives toward contraceptives influence their recommendations and can either promote or discourage women's contraceptive decision-making (Fataar et al., 2022; Lince-Deroche, Hendrickson, Moolla, Kgowedi, & Mulongo, 2020). Previous research has shown HCPs have concerns around effective and consistent use of contraceptives among young women and adverse events for older women, which impacts their contraceptive prescribing practices (Fataar et al., 2022). Provider behaviour change frameworks acknowledge the need to better understand providers’ underlying attitudes, biases and values, as well as the structural environment in which they work to positively influence the quality of services, enhance client experiences, increase demand for services and increase uptake of commodities or adoption of healthier behaviours (Breakthrough RESEARCH, 2019; Kalamar, Oyedokun-Adebagbo, & Reichenbach, 2023).

South Africa has the largest HIV epidemic in the world (UNAIDS, 2023), which has a significant impact on the health care system and its workers (Pleaner et al., 2023). Staff shortages, stock-outs and lack of training have contributed to inadequate counselling for sexual and reproductive healthcare, including for contraception and HIV prevention options (Lince-Deroche et al., 2020; Milford et al., 2018; Pleaner et al., 2023). There are disparities in the knowledge and awareness of new HIV prevention options among HCPs, often most pronounced among providers who see HIV-negative individuals. Support for PrEP and commitment from HCPs influences both access to PrEP and adherence (Pilgrim et al., 2018). Provider bias against prescribing PrEP to adolescent girls and young women presents an additional challenge to serving this population (Nyblade et al., 2022). Although contraception is available at low or no cost at public facilities in South Africa, over 50% of pregnancies are reported as unintended, with little change in fertility rates or contraceptive use indicators over the last decade (Chersich et al., 2017). There is a strong preference for injectable contraceptive options among HCPs in South Africa (Lince-Deroche et al., 2020) that potentially limits women's access to a menu of options and method choice. These perspectives are compounded by a host of system-level considerations and long wait times at clinics, which impact clients’ care (Kriel et al., 2023).

Previous findings underscore the significance of consulting HCPs throughout the drug development process to inform provider training, counselling and product introduction strategies for new health technologies and allow for successful product introduction (Brady & Manning, 2013; Pleaner et al., 2023). To address this, we sought to gain a granular understanding of HCP views about the concept of the DPP.

An existing framework for the DPP highlights the need to address three interrelated domains – **user-centred factors** (e.g. life stage, risk perception, attitudes, social norms), **healthcare provider-centred factors** (e.g. provider knowledge, counselling practices, service delivery environment) and **product-centred factors** (e.g. dosing regimen, formulation, regulatory and policy context) – for development and introduction (Friedland, Mathur, & Haddad, 2021). Situated within socio-ecological contexts, these factors aim to ensure informed choice, acceptability, and effective use, while facilitating integration of HIV and family planning (FP) services and serving as a model for future multipurpose prevention technologies (MPTs). Building on the DPP and provider’s behaviour change frameworks, we explored three HCP dimensions: (1) perspectives of the product and its attributes; (2) knowledge, attitudes and system-level considerations for the DPP and (3) experiences and opinions about end-users and their ability to effectively use the product. We conducted this study with HCPs in Johannesburg, South Africa, an area that continues to bear a disproportionate burden of HIV and other overlapping health concerns. Through this investigation, we hope to elucidate key considerations to ensure effective introduction and implementation of quality DPP services when this innovative product is available.

## Methods

### Study design

We implemented a cross-sectional qualitative study as part of a multiphase acceptability study assessing the DPP. Using purposive sampling, we conducted in-depth interviews (IDIs) with different cadres of HCPs (nurses, counsellors and a clinician) working in FP or HIV prevention and care sites. The site investigators and community liaison officers worked with local public health clinics to identify HCPs to interview.

### Study site and population

We interviewed HCPs in Hillbrow, an urban area of Johannesburg with a clustering of government facilities, health services and residential areas. As of 2020, Johannesburg had an overall HIV prevalence of 13%, which was highest among females of all ages: 28.3% for 25–49-year-olds, 15% for those 50 and above and 9% for 15–24-year-olds (City of Johannesburg, 2020). PrEP is available, and uptake has been increasing in recent years, with national uptake at 450,606 people as of December 2021 (Department of Health, 2022). In South Africa, 19% of females aged 15–49 who want to avoid pregnancy have an unmet need for modern contraception (Guttmacher Data Center, n.d.). Over 60% of sexually active women use a method of contraception, and ∼7% report using COCs (National Department of Health & ICF, 2019).

### Data collection procedures

Researchers approached HCPs and invited them to be interviewed. There was no screening procedure, per se, other than to interview representatives of different health care cadres at a mixture of family planning and HIV facilities. We had no *a priori* requirements for representation by sex assigned at birth, gender identity or age. HCPs who agreed to an IDI were interviewed by a qualitative researcher at a mutually convenient time in a private location in or near the HCP’s health facility.

We based the interview guide on the framework for DPP product development and introduction that emphasises the importance of product, provider and end-user-related characteristics and how their interrelation synergistically influences product acceptability, intention to use, uptake and use [23]. We explored HCP perspectives about DPP product attributes, how the DPP might meet their clients’ needs, factors that would influence DPP uptake and use, what HCPs would need to be able to provide the DPP and service delivery suggestions. Community advisory board members provided input on the interview guide before implementation. IDIs were conducted in English, isiZulu, or a combination of languages as selected by the HCPs. IDIs lasted approximately one hour, were audio recorded with HCP permission, transcribed and translated into English (if needed). Potential participants were told that being interviewed was voluntary, that their opinions would be kept confidential, that they could choose not to answer any question that made them feel uncomfortable and that they could end the interview at any time. We interviewed 17 HCPs between December 1, 2020 and March 31, 2021.

### Analytical approach

We used a thematic content analysis approach to analyse the data based on the framework for DPP introduction (Friedland et al., 2021). Data included interview transcripts, debriefing reports prepared by the data collection team and analysis memos created during data collection. We developed a code book based on the study objectives, the DPP framework and emergent themes from the data. We coded the transcripts using NVivo qualitative data analysis software (Version 1.7.1, QSR International). For quality control and to ensure reliability among the coding team, we double-coded approximately 10% of the transcripts. Thereafter, we reviewed and summarised each code report and grouped findings by perspectives about the product, end-user needs and provider and health system considerations, per the DPP framework. Weekly team meetings were used to discuss and resolve disagreements in coding, review code report summaries and discuss findings.

### Ethics

The study protocol was approved by the Population Council Institutional Review Board (New York, United States) and the University of the Witwatersrand Human Research Ethics Committee (Johannesburg, South Africa). All participants provided written informed consent before being interviewed and received the equivalent of ∼$10–20USD to compensate them for their time, according to local ethical guidelines.

## Results

HCPs were recruited from FP or HIV service delivery sites, including nurses, counsellors and a clinician (see Table 1). Participants ranged in age from 26 to 71 years and had been in their positions from 5 months to 20 years. We did not analyse data by sex (assigned at birth) because only two of the 17 HCPs were male. In general, we did not detect differences by HCP cadre or type of service delivery setting and present emerging perspectives from across the study participants. When there were differences by cadre or service delivery setting, however, they are indicated.

**Table 1.** Participants by site and role.

Type of heath service provision site	FP Site	HIV Site	Totals
Clinicians		1	1
Nurses	4	4	8
Lay counsellors (incl. HIV counsellors)	4	4	8
Totals	8	9	17

**Figure 1.**
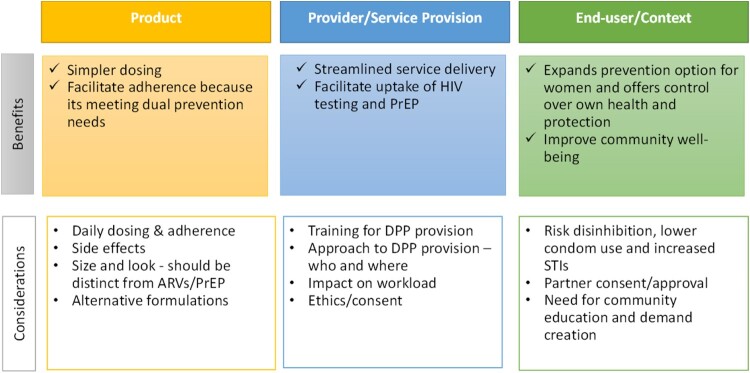
Summary of health care provider perspectives about the DPP, DPP service-provision, and DPP use (*n *= 17, South Africa).

Overall, all HCPs were enthusiastic about the DPP. HCPs in both FP and HIV service settings saw potential advantages of the DPP for individual clients and for service provision, overall. Simultaneously, they noted potential drawbacks about specific product attributes and clients’ ability to use the DPP effectively. Figure 1 provides a high-level summary of HCP perspectives on advantages and considerations for the DPP as related to the DPP product characteristics, service provision related to the DPP and end-user characteristics and context that may influence uptake and use of the DPP.

### DPP product characteristics

HCPs highlighted several critical considerations for the successful introduction of the DPP, which emerged across four sub-themes: dosing regimen and adherence, side effects, size and appearance of the pill and alternative formulations.

#### Dosing regimen and adherence

HCPs consistently emphasised that the convenience of a single-pill regimen was a major advantage of the DPP, likely to improve uptake and align with client preferences. Most HCPs thought that the main advantage of the DPP was the convenience and ease of use associated with taking a single pill. They felt that clients would be attracted to this method because taking only one pill would be easier to remember, would improve uptake and would align with client preferences for not using too many pills.

It is a great idea, it can work because taking PrEP and family planning, two tablets which might be taken at different times, so it means that they just take one pill, so it is easy to use more convenient. [Nurse, HIV site]

This product will have a positive impact because what I found very scary was that the ladies would use family planning methods but not protect themselves from HIV. I think having the DPP is the best method that will change people’s opinions about contraceptives, and their compliance will improve. I even believe that the ones that were not using contraceptives will use it for the benefits of PrEP. Not everyone is using contraceptives but because this will give them PrEP, a large number of people might come for the DPP. [Nurse, FP Site]

One provider felt that although the DPP might be difficult for people who are not used to taking COCs, the double benefit of the DPP would make the compromise of needing to take a pill every day worth it.

The only people that I think will have difficulty with the DPP are the ones who are not used to taking pills like the ones used to injectable, maybe adjusting might be a problem for them, but looking at the benefits we might have to compromise because the DPP have a double benefit. My clients are more matured and literate so this will not be difficult for them at all. [Nurse, FP Site]

Concerns about adherence were based on experiences with clients ‘defaulting’ on antibiotics, COCs, HIV treatment and the challenges of trying to get someone to be adherent to a prevention treatment. One HIV counsellor explained:

 …  if it was easy to take medication every day, we would not be coming across such people who are having a high viral load such people that are defaulting treatment. The reason why we are having such cases – it's because it is not easy. Especially the people who are taking medication for HIV, they know that they are HIV positive and in order to be virally suppressed they must take their medication. Here you are taking preventing something that is not there. So, chances of you being committed, being devoted are very slim because you know that you are not HIV positive or pregnant, you are just preventing it. [Counselor, HIV site]

HCPs were particularly concerned that younger women would not adhere to a daily preventative regimen.

A challenge would be that adolescents are not keen on taking something that they must drink every day. for a long time, that will be a challenge. [HIV counselor, FP site]

#### Side effects

There were concerns about side effects, with many HCPs emphasising that education on side effects will be the most vital information needed for HCPs in order to feel comfortable providing the DPP. HCPs noted that most women are used to COC side effects, which are considered less severe than other contraceptives like injectables or implants. Some HCPs were concerned that combining PrEP and COCs would amplify side effects.

I just hope that they [women] won't have side effects because if it [DPP] causes more side effects it will cause problems because of heavy side effects from Truvada … so I am just worried if it has many side effects, people will reject orals [contraceptive pills] they will go to other methods. [Nurse, FP site]

Many participants connected their concerns about side effects to the impact on adherence, as noted by an HIV counsellor at an FP site who said, *‘Side effects are problematic because they make women not adhere’.* HCPs specifically mentioned that adherence to the DPP would suffer if clients experienced side effects like headaches, weight loss, irregular or heavy bleeding, drowsiness, rashes, nausea or the need to return to the clinic frequently for side effect management.

One provider emphasised that because the DPP is for HIV prevention, not treatment, women would be less likely to tolerate side effects:

I think some people end up disengaging on [ARV] treatment because of side effects. Now that you know you [are] not HIV positive, you are not pregnant, you have to bear with the side effects, you will end up defaulting. You are not trying to achieve anything except that you are preventing yourself from encountering HIV and pregnancy. So, when you feel like you have side effect you will default because you can't have headaches, and lose weight, have nausea and continue like things are normal while they are not. You will rather say let me go back to using condoms and stop using this. [Counselor, HIV site]

However, a few HCPs noted that all medications have side effects, and these can be managed and tolerated with adequate counselling and monitoring.

#### Size and look of the DPP

HCPs emphasised that the DPP should not look like PrEP or ARVs and should not be too big compared to COCs. Several HCPs suggested it should be medium-sized – like a ‘Panado’ tablet (a common over-the-counter pain killer) – and round, between the sizes of COCs and PrEP.

It [size] is going to be a problem because it is too big as compare them to contraceptive pill that we have, they might have a problem in swallowing it, not exactly swallowing but they will think it is too big. [Nurse, FP site]

#### Alternative formulations

Several HCPs liked the idea of combining contraception and pregnancy prevention into one product but cautioned that a daily oral pill may not be right for all women. Injectables were the alternative regimen mentioned the most often for both convenience and efficacy. One participant also mentioned that a chewable tablet might be nice.

I have a suggestion that in future you should also create medication that is injectable, not only pills, because most people do not like pills because you have to take them daily at the same time. [Nurse, FP site]

I just think we need everything available some will stick for some people and the other method will stick by someone else. But I do feel that the more options I have as a clinician to offer someone the better chance of me being able to say if I am looking at prevention, I could be able to provide that. So, in theory I think is great to combine the two meds it makes sense, absolutely. [Clinician, HIV site]

### Service provision for the DPP

HCPs highlighted both enthusiasm for the potential benefits and practical considerations for successful implementation of the DPP. Key sub-themes included: anticipated advantages for clients and service delivery, training and information needs for providers, preferred approaches to distribution and counselling, implications for workload and ethical considerations related to consent.

#### Streamlined service delivery for clients

HCPs noted that the DPP would likely save their clients time and money. Access to a product that serves two health needs means clients only need to go to one point of service.

[DPP] will be beneficial because I won't be having to tell people to go to family planning and you come back after this day, you come for PrEP. So, for me DPP will make things easier because now the person is saving time. Imagine having to come for family planning maybe after two months or you are on the 3-month injection and I’m saying also you must come for PrEP because your window period ends on the 2nd month. You are coming to the clinic like nobody's business, you are up and down, so it is reducing that time you are spending at the facility for different services. [Counselor, HIV site]

#### Facilitate uptake of services

HCPs suggested that the DPP will result in more women getting tested for HIV and becoming *aware of* their HIV status.

What I would say about the DPP it is a really good drug and it will help a lot of women and it will attract women to come to the clinic and it will give them an opportunity to know their status. [Counsellor, HIV site]

#### Provider training for DPP provision

HCPs listed the types of information needed to adequately support clients with decision-making around the DPP (see Table 2).

**Table 2.** DPP product information requested by healthcare HCPs.

Product efficacy/level of protection against HIV and pregnancy offered by the DPP. Regimen and APIs included in the product. Eligibility criteria and any differences in prescribing pills to different age groups or people with certain health conditions. Lab and clinical procedures required before prescribing DPP. Side effects: anticipated common side effects (e.g. to body/ weight changes, nausea, vomiting), as well as rare/serious potential side effects and counselling and care guidance for common and rare side effects. Timing and flexibility in product use window: how to deal with missed pills and the window within which the pills still offer protection. Tips to facilitate swallowing the DPP. Detailed information on drug interactions. Information on DPP and its impact on return to fertility after the cessation of use.

HCPs confirmed that the successful introduction of the DPP would require detailed in-service training about the product and its use, and to help dispel any myths and misconceptions. A few HCPs cited limited opportunities for in-service training at their facilities and explained that sometimes they relied on social media, internet resources and advertisements (on TV, billboards, or flyers) as a source of information on new products.

Support, we should be trained and well informed about PrEP and contraceptives … because clients are quick to make up their own theories. think about the [contraceptive] implants, how they were quick to formulate myths and misconceptions. We need training and information to even address those myths because myths will be there. [Nurse, FP Site]

One provider explained that the training on DPP needs to be provided broadly within a health care setting because nurses often rotate within departments of a clinic and across local clinics.

 … at nursing we rotate the department, you can teach me but end up working there for 2 months then I rotate to another department, then the sister[nurse] who comes here has never had in service training. [Nurse, FP site]

#### Approach to DPP provision – where?

Participants unanimously suggested providing the DPP in primary health clinics and, although they were open to other options, opinions were more mixed on distribution via mobile clinics or vans, community-based organisations, schools, individuals’ homes or pharmacies. Table 3 summarises provider perspectives on the advantages and disadvantages of suggested locations for DPP service provision. The suggested provision locations are listed in order of priority, noted by participants.

**Table 3.** Potential DPP delivery location and advantages and disadvantages of each.

Location	Advantages	Disadvantages	Illustrative Quotes
Clinics	Women’s familiarity with clinics Exist in every community Free or low-cost service provision of COCs and PrEP (though not necessarily by the same provider) HCP and clinic capacity and capability (e.g., to treat side effects, conduct medical eligibility tests) Provision through FP clinics gives experience in contraceptive counselling and provision to reach sexually active women	Negative provider attitudes, especially towards youth clients (Nurses can be mean, have “*bad attitude*”) HCPs have limited time for detailed counselling Long queues Staffing shortages Loss of confidentiality Drug stockouts	*‘I think the family planning clinics should provide this DPP to the clients … ’*. [Nurse, FP site]
Pharmacies	Pharmacists are well-educated More privacy than clinics No queues Avoid nurses with bad attitudes	Costly for clients	*‘Maybe Pharmacies like Clicks [name of a local pharmacy chain]. I think they will also be able to provide. Because it is going to be easy to access it. … There will not be queues, like family planning clinics … they will just collect and go.’* [HIV counsellor, FP site]
Community-based Organisations	Team building mentality Youth-friendly Easier access than clinics	Need properly trained HCPs May be better for resupply vs initial provision	*‘Okay, I think those ones [community-based organizations] will be fine too … so you would see that a lot of youth like those things so they would go there, so having those it would be easier for them to be recruited and come to get the pill … ’* [Nurse, FP site]
Mobile vans	Convenient Reach more people/expa coverage Can reach rural areas	Difficult to start a drug there because you need trained people to prescribe a drug	*‘You see with mobile vans … I think those will work better in the rural areas, because the houses are dispersed so I think it might be better that way.’* [HIV counsellor, FP site]
Schools	Access to sexually active youth Many schools already provide contraceptionthrough school nurses	DPP provision could lead to perceived encouragement of sex and promiscuity Shortage of nurses/qualified people at schools to provide Need parental agreement to provide DPP at schools	*‘It [offering DPP in schools] will depend on the parents so that it does not come across as If we are encouraging them to have sex.’* [HIV counsellor, FP site]
Home-based provision	Convenient Time-saving – women do not have to spend a long time queuing at clinics	Expensive for the government to provide this way Lack of confidentiality/ privacy – inadvertent disclosure of DPP use could increase the risk of domestic violence	*‘It [providing DPP at people’s homes] will need a lot of money for the government, that’s my opinion, because they will need to employ more nurses.’* [Nurse, FP site]

#### Approach to DPP provision – who?

All HCPs emphasised the importance of proper counselling. Many participants indicated that a two-person team was needed; one to counsel and provide information to enable the person to make an informed choice, and a second person who was authorised to prescribe, counsel about side effects, provide detailed instructions on use, etc. Most people referred to nurse/counsellor pairs.

We will need our counsellors they play a big role because a professional nurse does not have all the time. We need the counsellor to do pre and post counselling. Most of the education is done by the counsellor, when the client comes to us, we assess and re-visit … . So, the counsellors are very important. [Nurse, FP site]

Nearly all participants said nurses would need to be involved in DPP service provision, some of whom emphasised the importance of having professional (registered) nurses to initially prescribe and dispense the DPP.

A nurse, health care provider [should provide DPP]. I think that will be much better because you know how to manage, you know what to look out for. The side effects, how to manage those. The examination of things, how to complete the file, it will be easy for someone who has done it before with other conditions to do it …  Counsellors will do the talking part, while the nurse will do the admin part and the vital signs. [Nurse, HIV site]

Doctors were identified as important players in DPP provision because they are trusted, are needed to address side effects, and have a better attitude than nurses.

Doctors and pharmacists [should provide DPP], you know, you talked about counsellors as well, I think them also. I think because those are the people that the community trusts and knows that when they go to them, they will not be judged and that they won't get bad attitude. [HIV counselor, FP site]

For participants who were specifically asked about pharmacists, all agreed that they should be involved in DPP provision. A few HCPs mentioned the importance of pharmacists for ensuring drugs were not dispensed past their expiry date or that they were not counterfeit.

When asked if there were other types of HCPs who should be involved in DPP service delivery, social workers, community health workers, health promoters and educators were cadres mentioned for education and counselling around DPP. Several HCPs said that everyone at the clinic should be educated about the DPP, from receptionists to data clerks tracking statistics and even the cleaners.

 …  I think by involving everyone from the person who is working at reception, maybe someone will ask the question from that very person and including the cleaners … people don't care what you know if they want to know something they ask everyone who is working in the health clinic. [Nurse, FP site]

#### Impact on workload

HCPs were mixed on whether the DPP would impact their ability to provide existing services effectively. Some HCPs felt that the DPP would reduce workload and that even though more clients might come to the site because of interest in the DPP, it would not disrupt their ability to provide services.

The only effects that we going to have is having more people on PrEP, there will not be any disturbance or anything because already we are giving oral contraceptives, we are doing ARVs, we are doing the PrEP although we have very few people on PrEP … . [Nurse, HIV site]

I do not think it will affect existing services because … It will be integrated in the family planning set up, … , those who wants pill you counsel them, you educate them, you tell them about this new pill, the dual prevention pills the advantages and the side effects, and the final choice is theirs. [Nurse, FP site]

However, other participants were concerned about existing provider shortages being exacerbated with the introduction of a new product that requires education and counselling.

We will be short staffed because now we will have more nurses dealing with the DPP in our facility. We need to get other nurses to come to help with the initiation of DPP because it is just not a pill that you give and say go home and take it, you will have to explain and provide proper counselling to the patient and explain everything including the side effects and the benefit of it. More nurses will be needed to deal with all that, and one to deal will side effects should the patient experience them. [Nurse, FP site]

I think there will be many people coming for the DPP than other services hence services like Family Planning will get stuck. They will be so many of that, the clinic nurses will end up giving them priority because the DPP queue will be so long that it affects other services … women coming for TOP [termination of pregnancy] services will be affected as well because they need to be tested before they go. [HIV Counselor, FP site]

#### Ethics and consent

HCPs generally agreed that *‘everyone has the right to access healthcare facilities … ’ [HIV counsellor, HIV site]*. HCPs distinguished the need for parental consent for young clients between the ages of 12–14 and those who are 15–17 years old. For those under age 15, a major concern was assessing the maturity level of clients and the need for parental consent if they felt that the young client did not understand the information being provided. HCPs further noted that if the parents were involved, they could also support their children to use services or adhere to care.

It is the consent part, having consent from the parents, as I said it before that it will help to involve the whole community and the parents will understand that we have these services for them and their kids because we want to avoid the kids from hiding that they are using these services. Parents also need to be the ones who encourages their children to use these services so that they do not get HIV. It will make it easier for us as well to provide these services to their kids. [Nurse, FP site]

HCPs expressed confusion and frustration due to inconsistencies in national regulations and guidelines between PrEP (recommended for those 15 years of age and above) and reproductive health care provision (for those aged 12 and above) related to consent for care for minors.

It's so strange how the government says a 12-year-old can sign for themselves when they want to abort, but a 16-year-old who is pregnant cannot sign themselves in for theater for a caesarean when giving birth. So those are the things that we are facing here. [Nurse, HIV site]

A few HCPs noted that despite the need for parental consent, they felt compelled to care for young clients as they did not want to fail them as HCPs.

 … as much as we know that mothers need to give consent, we need to take care of the patient as well because they come to the clinic to get our help because at home there is no space for them to talk about such things, for us is to provide the services. [Nurse, FP site]

Some HCPs talked about the use of consent forms as a way to protect themselves against clients or the clients’ families who may object to the care provided. While HCPs did not give specific examples of legal challenges or conflicts they faced with clients, a counsellor working at an HIV service site noted that *‘consent should be issued to protect everyone’* – meaning both the clients and the HCPs.

Ensuring that they sign a consent form so that when they face these side effects, they don't just come and say, no you gave me the wrong medication and it is doing this and that to me. You are the one that is making me sick. [Counsellor, HIV site]

### Uptake and use of the DPP

HCPs noted several interrelated sub-themes when asked about their perspectives on uptake and use of the DPP. These included perceived benefits for women's autonomy and expanded prevention options, anticipated community-level impacts and concerns about behavioural risk compensation such as reduced condom use. Providers also discussed gender dynamics – particularly male partner influence – alongside the critical need for community education and demand creation to support informed uptake.

#### Expanded prevention options for women

HCPs felt the DPP would expand prevention choices for women, allowing them to protect themselves from unintended pregnancy and HIV. Some noted that the DPP may be particularly relevant for women in serodiscordant relationships or women in relationships where they could not negotiate condom use.

… it's [DPP] also helping women to take power over their lives, over their bodies. [Counsellor, HIV site]

Let us say it happens that a woman gets raped, and she is using these preventatives methods, risk is high that a woman can be safe physically by not getting pregnant, by not getting HIV. It is a good pill but the part of having them both together, it is something else. [Counsellor, HIV site]

One provider also noted that the DPP would replace the placebo pills in the contraceptive regimen with something that women need.

 … so the 7 days of extra PrEP [chuckles] – I never thought of it – is a good addition because, we don't need the extra hormones, but we need the extra 7 days of PrEP, it is a good combination. [Nurse, FP site]

#### Improve community well-being

HCPs felt that the DPP could help reduce HIV burden in the community, as well as reduce the rate of unplanned pregnancies and associated abortions, maternal mortality and morbidity.

It is a good intervention. People have died from HIV. Girls have had abortions, and some have died because of unwanted pregnancies. [Nurse, HIV site]

A counsellor noted that they felt girls are more interested in preventing pregnancy than HIV, and with the DPP, they could do both and lower pregnancy rates in the community.

This DPP … has a lot of advantages because when you check like young girls especially, … they are more interested in preventing pregnancy than HIV. So, when there is this one pill, they are going to use it to prevent a lot of things that is going to decrease the stats of HIV, it's going to decrease the stats of pregnancy. [Counselor, HIV site]

Another nurse who currently works at an FP service site noted that the DPP could potentially benefit a lot of people, especially young people who have not yet been exposed to HIV. She went on to note that this in turn ‘*will be reducing HIV as a pandemic in the community’ [Nurse, FP site].*

#### Fear of increased risk behaviours and reduction in condom use

Throughout the interviews, there was a tension between the benefits of having more clients protected versus concerns that the DPP would lead to an increase in risk behaviours and reduced condom use. HCPs expressed worries that the DPP could contribute to women—especially younger women—being more promiscuous and less likely to use condoms. Some noted that they thought women were more worried about pregnancy than HIV; therefore, contraceptive use already contributed to reduced condom use. HCPs also raised concerns that if the DPP led to a further reduction in condom use, then there would be a rise in STIs.

after that they [adolescent girls] will have reckless sex because they are using DPP, do you see why I am worried … ** **that will give them the authority not to condomize, to go all over [to be promiscuous], to be reckless in their sexual activities with the knowledge that they are protected. [Nurse, FP site]

People are going to be encouraged to have sex without a condom, because I know this thing prevents HIV and I know I won't fall pregnant [Nurse, HIV Site]

An HIV counsellor was concerned that even mentioning the DPP to a client could make it seem as though she was encouraging her client to have sex.

You see it's like now to me it would be like you saying it is okay to have a boyfriend, it is okay to engage in sex as long as you are protecting yourself, that's one of the challenges. [Counselor, HIV site]

#### Male partner consent/approval

HCPs felt that the DPP would allow women to protect and take care of themselves and not rely on male partners. HCPs noted that for married women and women in relationships, their partners’ desires were a key challenge for prevention behaviours. With contraception, women sometimes needed to hide their contraceptives because men were unsupportive of contraception use or unwilling to use condoms. Similarly, disclosure and lack of open communication about HIV ere a challenge between partners. Within this context, HCPs felt that the DPP could be useful for women who are unable to use or discuss protection with their partners.

 … there are women who cannot negotiate for condom use and those who can negotiate and stand for themselves, but most of them cannot, and their men have the final word. So, this DPP will help those who cannot negotiate for condom use, because same men will not have a problem with their women taking DPP even though they do not want them to use condoms or even if they [women] do not tell their men they can just take their DPP and get protected … .so if we give them DPP it will empower the women who depend on their men for financial support, who have the final say on everything. [Nurse, FP site]

#### Need for community education and demand creation

HCPs stressed the importance of educating the community and emphasised the need for specific campaigns about DPP.

I think maybe going out from the clinic to the community to tell them about the DPP and going to schools as well to provide information to the youth and the teams and tell them more about DPP and where they can get it, how to access it, yes. [Nurse, FP site]

How do we create demand for any other prevention thing? … just a lot of outreach, a lot of community-based stuff, DPP days, that stuff, a lot of education around it …  [Counsellor, HIV site]

One provider highlighted the importance of communicating effectively and making it clear that the DPP includes the same drugs that clients are already used to.

The campaign – let's educate them and show them the drug and clarify to them that this is not the new drug. It is the drug that they already know … . People get scared of change if it's not communicated properly. … It is so important to make them understand that the pill they already know and [are] taking … .. [Nurse, FP site]

## Discussion

Our qualitative exploration of the DPP in South Africa provides insights from healthcare providers for the burgeoning field of MPTs. This is one of the first studies to capture family planning and HIV providers’ perspectives on the DPP currently under development, and it aligns with a rapidly evolving global health landscape emphasising integrated solutions and services (UNAIDS, 2025). Recent syntheses further underscore that MPTs can streamline access, reduce product burden, and catalyse integration of HIV, STI and contraceptive services across women's life courses, bolstering motivation and acceptability when multiple needs are addressed in a single product (Bershteyn, Resar, Kim, Platais, & Mullick, 2023; Friedland, Thurman, Nuwagaba-Biribonwoha, & Malcolm, 2024). HCPs in our study were supportive of this innovative product and felt that it would both fit an unmet need and expand the range of prevention options they could offer their clients, thereby improving sexual and reproductive health outcomes. HCPs also noted key considerations related to the product, its provision, and its use.

HCPs were enthusiastic about the convenience of combining HIV and pregnancy prevention into a single pill and felt that it would facilitate uptake and adherence, especially among women who were already using either COCs or PrEP. Their positive assessments are consistent with broader evidence that end users and providers favour discreet, user – controlled options – particularly when these products offer dual or multiple indications (e.g. HIV and pregnancy) (Dandadzi et al., 2025; Friedland et al., 2023). Programmatic guidance and advocacy from global stakeholders likewise frame MPTs as critical to meeting women's articulated preferences and improving equity in prevention access (Mgodi, Murombedzi, Murewanhema, Moyo, & Dzinamarira, 2025).

HCP concerns about the daily dosing regimen of the DPP reflected the challenges they have seen their clients face with daily oral medications (e.g. COCs and antibiotics) and HIV treatment adherence. A recent study with HCPs on four different potential MPT formulations, including an oral pill, found similar concerns about adherence (Lutnick et al., 2019). Similarly, a recent pilot study in Zimbabwe with an over-encapsulated DPP (a proxy for the current product in development) found high acceptability, but low adherence to both regimens (Mgodi et al., 2026). HCPs in our study wondered if alternative formulations, such as an injectable, could provide dual protection like the DPP. Future comparative research, including long-acting MPTs, can clarify how dosing and formulation might drive product preference and persistent use.

Another key consideration was around side effects, and the anticipated counselling and care demands around side effects. Prior research shows that perceived side effects can be a deterrent for uptake of new technologies (Chola, Hlongwana, & Ginindza, 2023; Jonas, Duby, Maruping, Harries, & Mathews, 2022). We found that HCPs were equally concerned about the potential for ‘dual’ or double the side effects, with the combination of the active pharmaceutical ingredients in oral PrEP and COCs. These concerns were driven by HCPs' experiences with PrEP provision and on counselling clients on managing the side effects associated with oral PrEP, which they felt were more than those of COCs or contraceptives in general. To effectively provide the DPP, HCPs will need detailed training about DPP attributes and anticipated side effects and adverse events to effectively counsel their clients (Segal et al., 2023).

HCPs in our study saw the DPP as a way of integrating HIV and other sexual and reproductive health service delivery. There has been a strong push to better integrate HIV services within FP or other health services, especially with recent cuts in the global and public health funding landscape. Specifically, they noted that the DPP should be provided at government-run public health clinics by FP providers. While there was a lot of discussion about alternative avenues for DPP provision, clinics made the most sense to participants in our study, as these are accessible in most communities, provide free or low-cost services, and are already frequented by women for other services. As women are most likely to frequent FP clinics, participants noted that FP clinics would be a natural point of introduction for the DPP. Unfortunately, while most health clinics offer PrEP and COCs, they are usually offered through separate departments. Evidence from Kenya and South Africa shows that integrating PrEP with existing SRH services for AGYW requires iterative learning, workflow redesign, and attention to staff motivation and resources; nevertheless, co-initiation and same-provider delivery can facilitate uptake and continuity (O’Malley et al., 2021). To date, however, there is limited delivery of PrEP outside of HIV clinics or departments in South Africa and specific regulations around who can prescribe PrEP and where – namely, doctors or nurses with comprehensive training in HIV treatment and care at HIV clinics and some community-based organisations can prescribe PrEP. PrEP is not yet accessible over the counter without a prescription, and while distribution through trained pharmacists has been under discussion, it is not yet implemented (Nelson et al., 2022). Thus, there is limited access to PrEP across sectors or other cadres of HCPs. This has clear challenges for the structural context within which DPP can be provided, both in South Africa and other places with similar regulations for PrEP provision.

Additionally, mirroring prior research, our study shows that provider biases, attitudes and perceptions are likely to play an outsized role in HCPs’ willingness to provide the DPP (Jonas et al., 2019; Nyblade et al., 2022; O’Malley et al., 2021; Pilgrim et al., 2018). HCPs in our study extensively discussed the judgmental attitudes of nurses at public clinics, particularly toward younger clients, and how this dissuades health service use at the clinics. In recognition of these biases, and because the DPP requires effective counselling tailored for different age groups, HCPs suggested alternate venues for DPP delivery, including pharmacies, community-based organisations and mobile clinics. They also suggested that paired teams of counsellors and nurses could effectively inform women about the product and support effective use. Further, to make clinics more receptive to younger clients, HCPs suggested a ‘whole clinic’ approach, wherein everyone at the clinic should be educated about the DPP, from receptionists to data clerks tracking statistics and even the cleaners. Similar ‘total clinic’ approaches are successful in addressing HIV-related stigma and facilitate client access to HIV services (Nyblade et al., 2020) and may be warranted for the DPP or other MPTs as well.

HCPs expressed confusion when navigating parental consent guidelines for different medical services for adolescents. For instance, the South African Children's Act allows HIV testing for children under 12 and *the* provision of contraceptives and condoms to children 12 and above without parental consent. However, with PrEP, South African guidelines recommend PrEP for clients aged fifteen and above. These regulations are further conflicted by social norms around adolescent sexuality, and HCPs' desire to meet the needs of their clients leaves them without clarity on how to provide services to adolescents. Jurisdictional guidance that further clarifies consenting for preventive services (including HIV testing, PrEP/PEP and contraception) and prioritises youth-friendly care will be useful for operationalising DPP in settings with mixed regulations (UNAIDS, 2022).

Ultimately, HCPs in our study felt that the DPP (and other MPTs) could empower women and girls and meaningfully expand choice for women. They suggested that the DPP would increase uptake of contraception and HIV prevention overall. HCPs felt that the DPP would be especially pertinent for women whose partners do not want to use condoms or contraception. However, HCPs also pointed to several potential challenges, such as the need for partner approval among married women and the need for community education about the DPP to dispel myths. Issues like partner approval are not specific to the DPP and have been noted in prior research as a challenge in contraceptive and PrEP uptake and effective use (Blackstone, Nwaozuru, & Iwelunmor, 2017; Chola et al., 2023; Jani, Mathur, Kahabuka, Makyao, & Pilgrim, 2021; O’Malley, Hawk, Egan, Krier, & Burke, 2020). HCPs pointed out that the DPP in and of itself would not negate the norms and pressures women experience within their relationships. Counselling women around disclosure related to product use and engaging men as partners may need to be considered, alongside community-led demand generation strategies for DPP provision (Dandadzi et al., 2025; Nyagah et al., 2023).

Our study is not without limitations. We conducted formative research about a hypothetical product that is not yet on the market. As the DPP becomes available, additional implementation studies will be needed to better understand HCP and client experiences with service provision in varied contexts. We did not see any major differences in HCP perspectives by sex; however, our small sample was predominantly female. The impact of training, background and sex on HCP perspectives on MPT provision should be explored in the future among a bigger sample of providers. Subsequent research may also want to explore HCP perspectives in areas with a different contraceptive method mix. Injectable contraceptives are popular in South Africa, for instance, and that may have influenced provider perspectives regarding a daily oral pill. Our study was conducted during the height of the COVID-19 pandemic, and thus, we had difficulty recruiting clinicians at public health facilities in our catchment area given the other burdens on their time. Further, while we made concerted efforts to recruit HCPs from public health facilities, these were located in Hillbrow – a densely populated urban area with some exposure to health research. Future studies may want to explore the particular roles/perspectives of clinicians, nurses and counsellors from urban as well as rural areas for DPP service provision.

## Conclusion

This was the first of its kind qualitative exploration about the DPP with HCPs providing HIV and FP services in public health facilities. HCPs pointed to several potential advantages of the DPP, and suggested enhanced use, streamlined delivery and meeting client needs. They also highlighted challenges related to the DPP (e.g. daily dosing regimen) and underlying social (e.g. partner approval) and structural challenges (e.g. negative HCP attitudes, potential provision only in HIV clinics). These findings deserve close consideration as DPP market access strategies and provider training materials are developed. Comprehensive approaches that strategically consider how the product is packaged and introduced, how it is provided, in what context and with what support – will be needed for DPP introduction.

## Data Availability

Sharing qualitative data, such as transcriptions from interviews, can potentially reveal sensitive information and identify individual participants. The potential for identification from interview transcripts is particularly high in our study, given the small population from which the participants were recruited. Any researchers who meet the criteria for access to confidential data with a reasonable request may contact the corresponding author.

## Justification

This is a collaborative project between two research teams based in the USA and South Africa. The list of authors reflects author contributions for the conceptualisation, implementation, analysis and manuscript preparation, as outlined in the author contributions.
